# A realist process evaluation of an intervention to promote competencies in interprofessional collaboration among interdisciplinary integrated care teams for older people: Study protocol.

**DOI:** 10.12688/hrbopenres.13729.1

**Published:** 2023-09-13

**Authors:** Deirdre O'Donnell, Emer Ahern, Carmel Davies, Aoife De Brún, Sarah Donnelly, Thelma Doran, Anne Drury, Nikki Dunne, Lillian Finucane, PJ Harnett, Reema Harrison, Deirdre Lang, Eilish McAuliffe, Mary McCarthy, Catherine McGuigan, Éidín Ní Shé, Gráinne O'Donoghue, Marie O'Shea, Apolonia Radomska, John Travers, Helen Whitty, Catherine Devaney

**Affiliations:** 1 School of Nursing, Midwifery and Health Systems, University College Dublin, Belfield, Dublin 4, Ireland; 2 The Office of National Clinical Advisor and Group Lead (NCAGL), Health Service Executive, County Dublin, Ireland; 3 School of Social Policy, Social Work and Social Justice, University College Dublin, Belfield, Dublin 4, Ireland; 4 Public and Patient Representative, Ireland, Ireland; 5 Unit 8, 4075 Kingswood Rd, Citywest Business Campus, Family Carers Ireland, Dublin, Dublin 24, Ireland; 6 National Clinical Programme for Older People, Health Service Executive, County Dublin, Ireland; 7 Australian Institute of Health Innovation, Macquarie University, Sydney, Australia; 8 Age Friendly Ireland, Shared Service Centre, Meath County Council, Buvinda House, Navan, County Meath, Ireland; 9 Graduate School of Healthcare Management, Royal College of Surgeons of Ireland, Dublin, Ireland; 10 School of Public Health, Physiotherapy and Sports Science, University College Dublin, Belfield, Dublin 4, Ireland; 11 School of Medicine, University College Dublin, Belfield, Dublin 4, Ireland; 12 Knowledge User (NCPOP) lead, National Clinical Programme for Older People, County Dublin, Ireland

**Keywords:** older people, interprofessional collaboration, team working, care integration, transitional care, coordination, professional development

## Abstract

**Background:** International policy is increasingly committed to placing interdisciplinary team-working at the centre of health and social care integration across the lifespan. The National Clinical Programme for Older People in Ireland has a critical role in the design and implementation of the National Older Person’s Service Model, which aims to shift the delivery of care away from acute hospitals towards community-based care. Interdisciplinary Community Specialist Teams for older persons (CST-OPs) play an important role in this service model. To support the development of competencies for interprofessional collaboration and an interdisciplinary team-based approach to care integration, a culture shift will be required within care delivery.

**Design:**This study builds upon a collaborative partnership project which co-designed a framework describing core competencies for interprofessional collaboration in CST-OPs. A realist-informed process evaluation of the framework will be undertaken as the competencies described in the framework are being fostered in newly developed CST-OPs under the national scale-up of the service model. Realist evaluation approaches reveal what worked, why it worked (or did not), for whom and under what circumstances. Three iterative and integrated work packages are proposed which combine multiple methods of data collection, analysis and synthesis. Prospective data collection will be undertaken within four CST-OPs, including qualitative exploration of the care experiences of older people and family carers.

**Discussion:** The realist explanatory theory will provide an understanding of how interprofessional collaboration can be fostered and sustained in various contexts of care integration for older people. It will underpin curriculum development for team-based education and training of health and social care professionals, a key priority area in the national Irish health strategy. It will provide healthcare leaders with knowledge of the resources and supports required to harness the benefits of interprofessional collaboration and to realise the goals of integrated care for older people.

## Introduction

International health system policy and operational models have increasingly identified interdisciplinary teamwork as critical for health and social care integration across clinical pathways ( Science Advice for Policy by European Academics, 2019; World Health Organisation, 2016). International evidence identified coordination, cooperation, and collaboration as key priority areas for designing and implementing integrated care models ( Burke *et al.*, 2021). In Ireland, health system reform underpinned by national policy has identified interdisciplinary team-based approaches to care integration as critical for enabling a case management approach to care integration for older people ( Department of Health, 2021). This team-based approach to integrating health services involves changing how health and social care is planned and delivered ( Barry *et al.*, 2021; Ní Shé *et al.*, 2020). Interdisciplinary community specialist teams for older persons (CST-OPs) have recently been introduced to support the delivery of the National Older Person’s Service model. These newly formed teams require support to develop new ways of working particularly in relation to interprofessional collaboration ( Ní Shé *et al.*, 2020; O’Donnell *et al.*, 2022).

Interdisciplinary team working is a core feature of common international models of older people’s health and social care and is considered critical for comprehensive geriatric assessment and care planning ( Ellis & Sevdalis, 2019; Ellis *et al.*, 2011). There has been some attention in the literature given towards the development of competencies for interprofessional collaborative practice in general health ( Interprofessional Education Collaborative, 2016; Schmitt *et al.*, 2011; Wood *et al.*, 2009). However, there is a knowledge gap concerning how team working can be enhanced and supported in older people’s care specifically ( Ellis & Sevdalis, 2019). The European Competency Framework for Health and Social Care Professionals working with older people outlines a minimum set of competencies constituting a common baseline for Health and Social care Professionals (HSCPs) ( Dijkman *et al.*, 2016); however, the framework does not focus on interprofessional collaboration and integrated care. Despite the development of the interprofessional capability framework for the prevention and management of frailty ( Roller-Wirnsberge *et al.*, 2020) there remains limited understanding of how meaningful interprofessional collaboration can be fostered, enhanced or sustained within an interdisciplinary team-based approach to care integration for older people.

The ECLECTIC project (Embedding Interprofessional Collaboration to foster Integrated Teamworking in the Care of Older People) sought to address this gap by co-designing a competency framework that provides practical guidance for building competencies for interprofessional collaboration in the context of older people’s integrated care ( O’Donnell *et al.*, 2021). The ECLECTIC framework outlines the core competencies necessary for interprofessional collaboration in interdisciplinary CST-OPs ( O’Donnell *et al.*, 2021; O’Donnell *et al.*, 2022). The National Clinical Programme for Older People (NCPOP) adopted the framework to support the clinical design and operational guidelines for CST-OPs established as part of the older person’s service model ( National Integrated Care Programme Older Persons, 2021). These CST-OPs will be supported by the NCPOP to deliver integrated services and pathways for older people with complex health and social care needs. The programme aims to shift care delivery away from acute hospitals towards community-based, planned and coordinated care ( National Clinical and Integrated Care Programmes, 2017).

The ECLECTIC framework was co-designed with healthcare professionals, from across thirteen disciplines involved integrating care for older people in community care settings. Five Public and Patient Representatives (PPRs) also joined the co-design team and were critical in the development and validation of the framework. Furthermore, in-depth qualitative research was undertaken with two interdisciplinary community-based teams integrating care for older people. The qualitative analysis was used to contextualise the core competencies described in the framework ( O’Donnell *et al.*, 2022).

**Figure 1.**
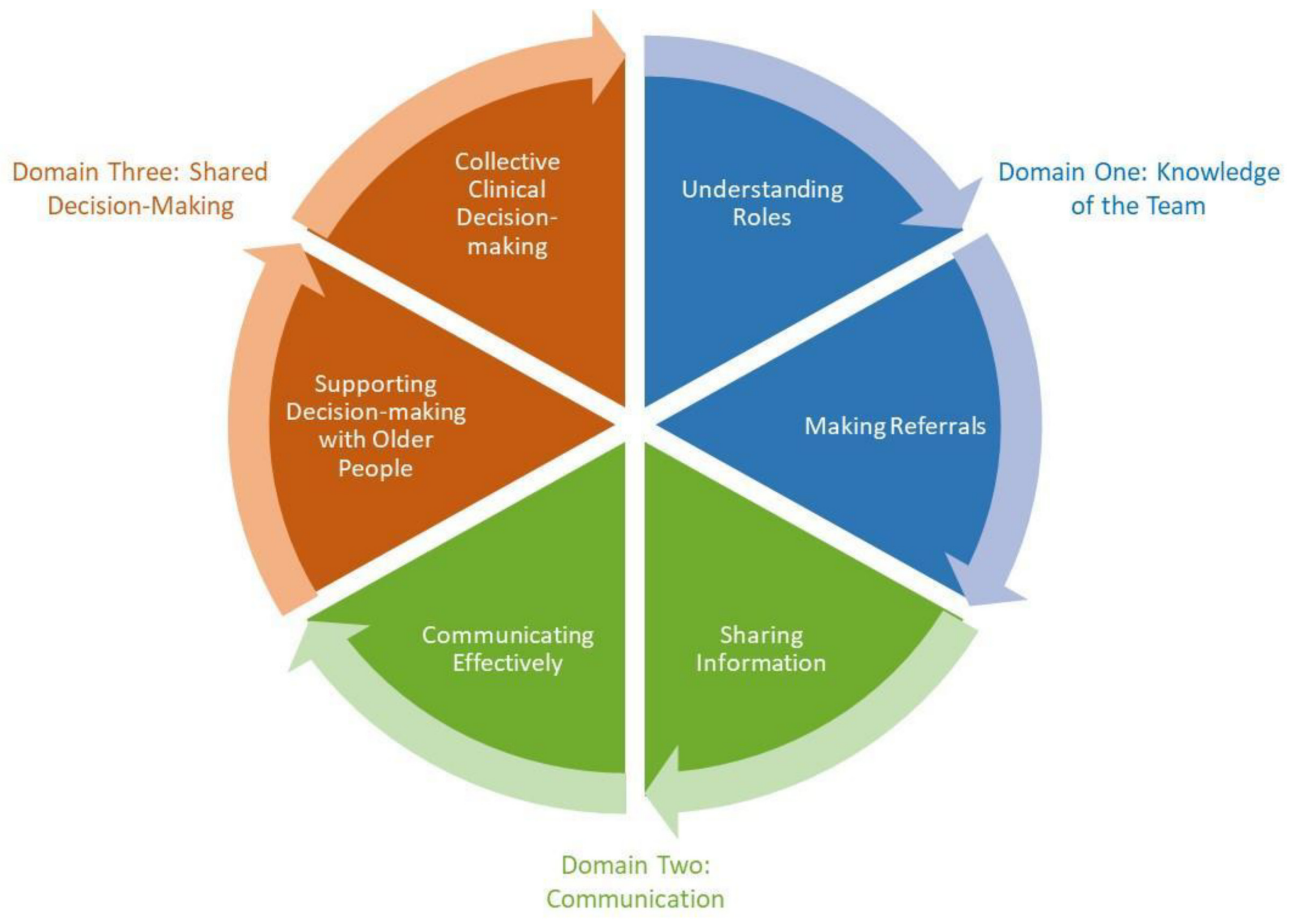
Three domains describing six competencies for proficiency in inter-professional collaboration within integrated care of older people ( O’Donnell *et al.*, 2021).

The ECLECTIC framework describes three domains of competence ( Figure 1). Each of the domains contain two competencies supporting interprofessional collaboration.

- Domain one- Knowledge of the Team. This domain includes the competencies, understanding roles and making referrals.
- Domain two- Communication. This domain includes the competencies, sharing information and communicating effectively.
- Domain three- Shared Decision-making. This domain includes the final two competencies, supporting decision-making with older people and collective clinical decision-making.

The six competencies are complementary, whereby proficiency in one support proficiency in the other. For example, understanding of team roles supports making effective referrals which in turn supports sharing of information.

The six competencies described in the ECLECTIC framework are aligned with elements of the European Collaborative and Interprofessional Capability Framework for Prevention and Management of Frailty ( Roller-Wirnsberge *et al.*, 2020). The external validity of the framework is further supported by Schmitt *et al.* (2011) and Greilich *et al.* (2023), who identified core teamwork competencies for interprofessional collaborative practice which are critical for the transformation of health and social care education and curricula. The co-designed framework aligns with these competencies by recognising the importance of creating a psychologically safe environment, shared team values, mutual respect, trust, understanding of roles and responsibilities and effective communication with patients, families and HSCPs. The ECLECTIC framework provides value to the European Capabilities Framework ( Roller-Wirnsberge *et al.*, 2020) and the competencies identified by Schmitt *et al.* (2011) and Greilich *et al.* (2023). The framework builds upon these models by providing practical guidance on how teams collaborating in caring for older people can enhance competence for effective interprofessional collaboration.

Non-technical skills are increasingly recognised as critical to effective interdisciplinary working ( Ellis & Sevdalis, 2019). In the ECLECTIC framework these skills are depicted as supporting shared and collective decision-making, effective communication, and successful alignment of disciplinary roles and responsibilities towards a common vision established by the team. The framework further outlines evidence-based processes associated with principles of good governance for sharing information as well as embedding new ways of working for referral pathways and care planning ( National Clinical and Integrated Care Programmes, 2017). Finally, and perhaps most importantly, person-centred values underpin the knowledge, skills and behaviours outlined within the framework. The will and preferences of the older person are depicted in this framework as being at the centre of effective interprofessional working necessary for care integration ( Science Advice for Policy by European Academics, 2019; World Health Organisation, 2016).

The proposed study will carry out a realist-informed process evaluation of how CST-OPs integrating care for older people develop core competencies for interprofessional collaboration through the adoption and adaptation of the ECLECTIC framework. The evaluation will focus on the competencies as they are fostered in the national scale up of the newly developed CST-OPs. The proposed realist evaluation will generate evidence regarding the outcomes associated with interprofessional collaboration in the care of older people. Furthermore, it will provide a nuanced realist understanding of the influence of contextual conditions in enabling or inhibiting mechanisms that foster, enhance, and sustain interprofessional collaboration. This explanatory theory will expand the ECLECTIC framework with an understanding of what works in supporting interdisciplinary teams to develop competency in interprofessional collaboration, for whom does it work, in what contexts, and how.

This understanding will underpin curriculum development for team-based education and training of health and social care professionals, a key priority area in the national health strategy. It will also inform future international research by providing a foundational theory to support the exploration of how interdisciplinary teams across multiple healthcare settings, specialities, and contexts can build competencies for interprofessional collaboration. In this way, the proposed project will provide a transformative direction for future international health service workforce development and the capacity building necessary to reform international health systems shifting from acute, episodic care to longitudinal community-based, coordinated and integrated care models.

### Study aim

We aim to evaluate the implementation of the ECLECTIC framework for the development of core competencies for interprofessional collaboration in CST-OPs.

The primary outcome of this project is to use the knowledge generated from the evaluation to underpin curriculum development for the education and training of health and social care professionals. Furthermore, the study will provide healthcare leaders with an understanding of how interprofessional collaboration can be fostered and sustained in interdisciplinary teams integrating care for older people. Leaders will gain knowledge of the resources and supports required to harness the benefits of interprofessional collaboration and realise the goals of care integration for older people.

Specific objectives are to:

1. Develop an expanded and enhanced ECLECTIC framework supported by an implementation strategy. The strategy will include operational guidance to support national implementation.
2. Develop a co-designed curriculum framework for interdisciplinary teaching and learning of competencies for interprofessional collaboration in the care of older people.
3. Develop learning resources for CSTs in collaboration with the Health Service Executive (HSE) and NCPOP that will enable team members to develop competencies for interprofessional collaboration. These resources will provide specific learning activities and materials aligned to relevant contexts-mechanism-outcome configurations and will be made available to all the CST-OPs supported by NCPOP.
4. Contribute to the theoretical understanding of what works (mechanisms) in building proficiency for interprofessional collaboration and under what circumstances (contexts) through the development of a middle-range theory providing a detailed explanation of the dynamic relationship between varying context-mechanism-outcome configurations.

## Methods

### Ethics approval

Ethical review and approval will be sought from the relevant governance committee once the case-study sites are identified. Furthermore, ethical exemption will be sought from the UCD Life Sciences Ethics Committee.

### Design and procedure

The study adopts a realist evaluation approach which is an interpretive theory-driven approach to evidence synthesis which uses multiple sources of evidence including published peer-reviewed studies, policy documents and grey literature. This evidence is combined with stakeholder theories and explanations of how interventions might work ( Kantilal *et al.*, 2020). The focus of realist evaluations is on ‘what works, for whom, under what circumstances and how’ rather than determining outcomes of interventions. This results in a series of evidence-based initial programme theories (IPT) in the form of statements that explain the mechanisms (M) and resources (R), generated within contexts (C) in response to interprofessional collaboration, that are thought to lead to enhanced care outcomes (O).

**Figure 2.**
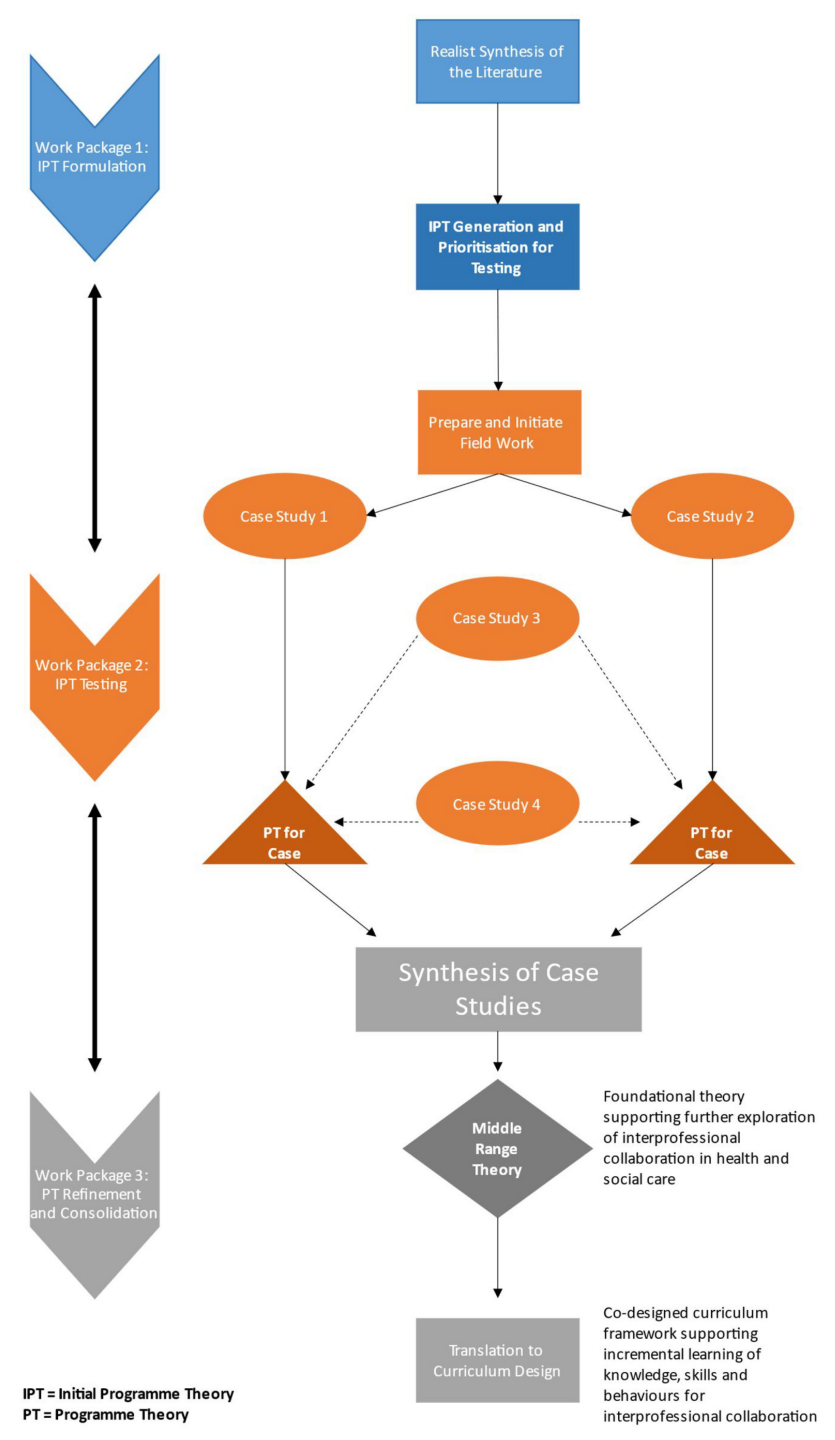
Overview of the three work packages.

The research design is mapped out over three consecutive work packages ( Figure 2). This will involve a realist review and synthesis of the literature in collaboration with four key stakeholder groups (HSCPs, operational leads, policymakers and clinical design managers, and public and patient representatives (PPRs)) to develop initial programme theories (IPTs).

The IPTs will be tested, refined, and consolidated through an in-depth exploration of the processes and experiences within four CSTs for older people who are selected as case study sites. This will involve structured interviews with team members, semi-structured interviews with older people and family carers, quantitative outcome measurement, observations of team meetings and document analysis.

This evidence will be synthesised and consolidated into a middle-range programme theory outlining a detailed explanation of the dynamic relationship between varying C-M-O configurations. The middle-range programme theory will identify guiding principles that can be applied in practice to support effective interprofessional collaboration in the delivery of quality care integration for older people. Translation of this theory into professional standards and guidelines for service design will contribute to ongoing international activity concerning health workforce capacity development and curriculum design for continuing professional education and training.

### Work package one: Initial programme theory formulation

**Figure 3.**
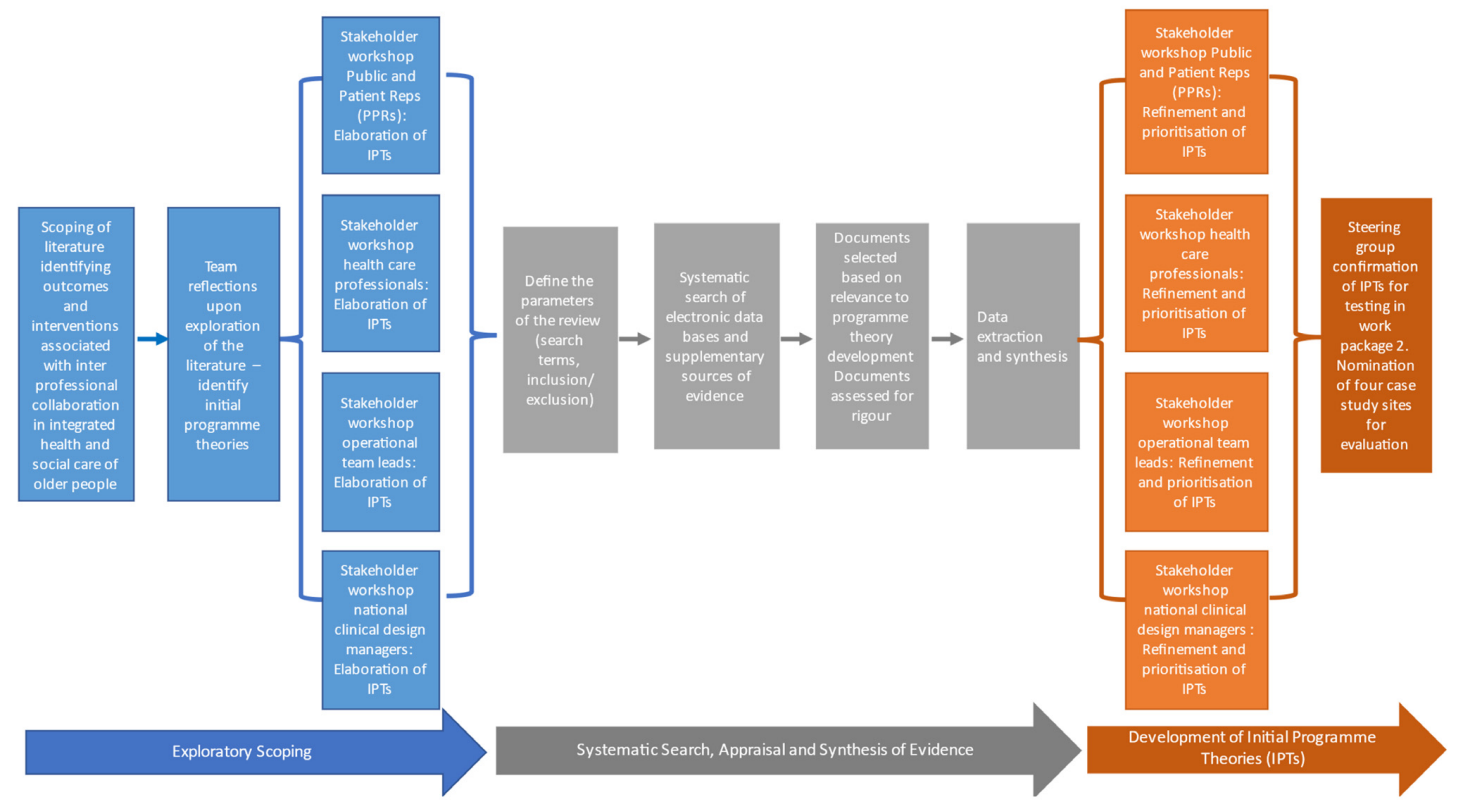
Overview of work package one.

Work package one involves a realist review of the international research evidence and local stakeholder evidence regarding what works and why in fostering competencies for interprofessional collaboration in CSTs for older people ( Figure 3).

Realist reviews are an interpretive theory-driven approach to evidence synthesis which uses multiple sources of evidence including published peer-reviewed studies, policy documents and grey literature ( Kantilal *et al.*, 2020). In keeping with the principles of realist methodology the evidence will also be combined with stakeholder theories and explanations of how interventions might work ( Pawson & Tilley, 1997). Three stakeholder cohorts have been identified as the following:

1. Health and social care professionals who are members of CST-OPs coordinating care for older people. A sub-group of this cohort includes operational team leads responsible for local governance of teams.
2. Policy makers and health care managers from NCPOP, the Health Service Executive and the Sláintecare Implementation Group. Furthermore, representatives from our collaborator nongovernmental organisations (Family Carers Ireland and Age Friendly Ireland) will be included.
3. Public and patient representatives (PPRs) of older people and family carers (our PPI advisory group).

This review will focus on generating initial programme theories (IPTs) for the contexts and the mechanisms that are associated with the outcomes of interventions targeting interprofessional collaboration ( *i.e.,* patterns of generative causation). These IPTs will support the evaluation of the implementation of the ECLECTIC framework within CST-OPs operating under the NCPOP (Work Package Two). The realist review will be guided by five steps adapted by Kantilal *et al.* (2020) which are categorised into an exploratory scoping phase (steps 1–2), a systematic search, appraisal, and synthesis phase (steps 3–5). The synthesised evidence will be reviewed and developed into initial programme theories.

***Exploratory scoping of the evidence***

**Define the review scope**

This will involve an informal exploratory scoping of the literature with a particular focus on interventions which support interprofessional collaboration within health and social care teams and the outcomes associated with interdisciplinary working in the integrated health and social care of older people. The primary focus of this scoping and exploration phase of the review will be to determine the scale of the body of evidence available for the realist synthesis and to help with the definition of terms as well as the specific realist review questions.

**Develop initial programme theories**

Realist programme theories are abstract descriptions of the mechanisms generated within contexts in response to interventions and how they are assumed to cause different outcomes. These theories illustrate the relationships between contexts, mechanisms and outcomes and are expressed as CMOC configurations ( Pawson & Tilley, 1997). We will develop IPTs from our team reflections upon the exploratory scoping of evidence. Furthermore, we propose to conduct four workshops with the key stakeholders (HSCPs, operational leads, policy makers and clinical design managers, and PPRs) to assist with the elaboration of IPTs to guide step three of the review process.

***Systematic search and appraisal***

**Search for evidence**

This step involves the identification of suitable evidence to test and refine the IPTs that emerged from the stakeholder consultations and the exploratory scoping of evidence. A systematic search of the following electronic databases will be conducted: Medline, EMBASE, CINAHL, SCOPUS, PsycINFO, and PUBMED. Search terms will be developed in discussion with the review team. Supplementary sources of evidence will also include reference lists from primary studies and systematic reviews, citations searches, stakeholder recommendations and steering committee recommendations. Purposive retrieval of evidence will be essential for the identification and inclusion of relevant grey literature, guidelines, policy, and standard documentation.

**Select and appraise evidence**

We will use systematic methods for study screening and selection following the PRISMA guidelines ( Shamseer *et al.*, 2015). Two reviewers will independently screen papers for inclusion/exclusion criteria first by title and extract and then by full text. Disagreements will be resolved by discussion with a third reviewer to ensure there is consistency in evidence inclusion. It is recognized step three (searching for evidence) and step four (evidence selection and appraisal) will be an iterative process and purposive searching for evidence may be necessary to elaborate and provide context for the emerging programme theories. Documents will be selected based on their relevance to the programme theory development. The documents will be assessed for rigour in terms of overall trustworthiness of the evidence. This will include an assessment of the transferability of the data, the dependability of the methods and the credibility of the findings.

**Extract and synthesise data**

Data will be independently extracted by two reviewers according to a bespoke data extraction form. The extraction form developed for this review will include information about the study aims, intervention design, study methods, participants, outcomes and measurement and information relevant to the emerging programme theories on context and mechanisms. As per the realist approach to reviews, data will focus on author explanations about how and why an intervention was assumed to have worked or not worked ( Kantilal *et al.*, 2020). Sections of the relevant texts will be coded inductively (through emergent codes) as well as deductively (using the emerging programme theory). The extracted coded data from different sources of evidence will be synthesised together under the domains of contexts, mechanisms, and outcomes. Focus will be given to identifying emerging patterns of relationships between codes and between coding domains. This will involve iterative and ongoing reflection and discussion among the review team.

***Development of initial programme theories (IPTs)***

Finally, four validation workshops will be held with key stakeholders (HSCPs, operational leads, policy makers and clinical design managers, and PPRs) to review, refine and prioritise the IPTs. This will result in a series of evidence based IPTs which are context specific theories in the form of statements which explain how and why teams build interprofessional collaboration and what the associated outcomes are.

### Work package two: Initial programme theory testing

**Figure 4.**
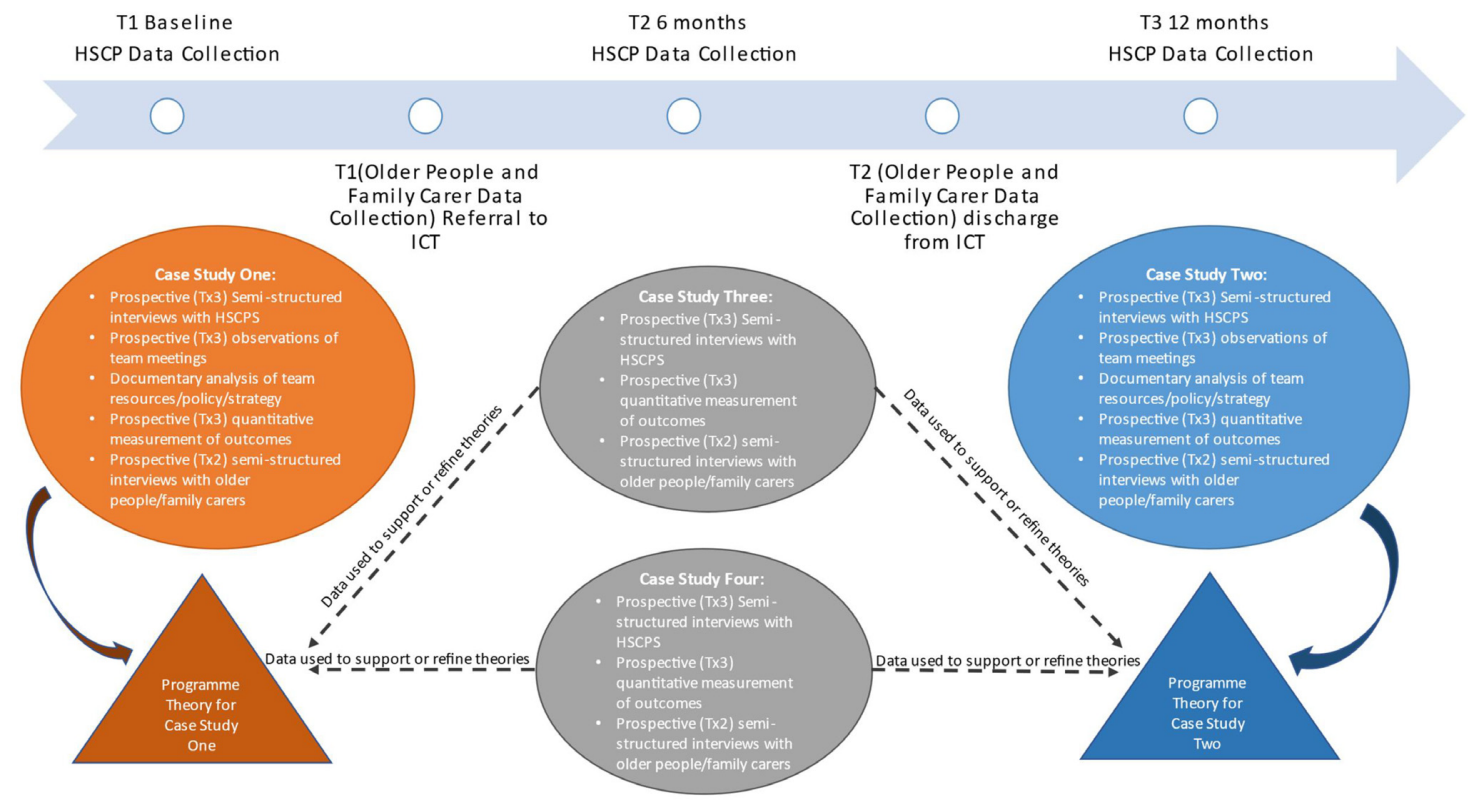
Overview of work package two.

The second work package will involve multiple methods to explore, evaluate and test the IPTs ( Figure 4). Prospective data collection will be undertaken within four CST-OP case study sites. The selection criteria for the four nominated teams will ensure the teams are representative of the complexity and diversity of programme CST-OPs in terms of geographical location (urban and rural), service area population density and cultural diversity as well as team compositions and stage of implementation (early adopters *versus* more established teams). Furthermore, case study site selection will be guided by the IPTs that were prioritised at the end of work package one for testing.

Due to the given complexity of the system in which the ECLECTIC framework is being implemented, each of the four teams will likely be at different stages in their development proficiency for interprofessional collaboration. This will provide additional nuance and understanding of the role of context in the realist evaluation. Two of the nominated teams will be identified by the research team in collaboration with the project advisors for the collection of extensive process data and a more in-depth realist evaluation. This evaluation will primarily focus on testing and refining the emerging programme theory. The other two teams will provide prospective qualitative data that can be used to contextualise and support the emerging theory.

***Data Collection: Four case study sites***

The following prospective data will be collected from the four nominated CST-OPs: 

**Structured interviews with team members**

Qualitative structured interviews will be conducted with members of each of the four nominated CST-OPs (N=24–32) at baseline (T1), six months (T2) and at 12 months (T3). The participants will be purposely selected to ensure representation from each discipline represented on the team as well as a support member such as operational manager (n=6–8 per team). We aim to interview the same team members at each time-point to enable prospective exploration of changes over time. The interviews will be guided by a structured format derived from the IPTs. The participants will review the IPTs and explore each of the configuration elements (context, mechanisms, outcome) from their own experience of interdisciplinary and interprofessional working. The participants will determine whether the interviews will be conducted either face-to-face or remotely using video conferencing technology as per their requirements and preferences.

**Semi-structured interviews with older people and family carers**

Prospective qualitative interviews will be conducted with older people and/or their nominated family carer who are receiving integrated care from one of the four nominated care teams. Interviews will be conducted in two waves: following initial referral/assessment and following discharge from the CST-OP. We will aim to prospectively interview 16–20 older people and/or family carers across four team sites at each time point (n=4–5 per team). The focus of these interviews will be to explore the participants’ care experiences and needs during their time with the CST-OP and to identify how needs change over time. The IPTs will inform the interview guide and will prompt discussion of the outcomes identified in the configuration statements from the perspective of older people and/or family carers. This discussion may also identify potential causal mechanisms for these outcomes. These interviews will be conducted face-to-face unless otherwise requested by the participant themselves.

**Quantitative outcome measurement**

The quantitative outcomes associated with interdisciplinary collaboration identified from the systematic scoping review in work package one will be measured at three timepoints (baseline (T1), 6 months (T2), 12 months (T3) as above). Each of the CST team members (N=52–55) will complete an online questionnaire measuring outcomes that have been adopted from the mechanisms for evaluating team competency in interprofessional collaboration described in the co-designed ECLECTIC competency framework ( O’Donnell *et al.*, 2021; O’Donnell *et al.*, 2022) as well as the HSE’s operational guidance for older person community-based multi-disciplinary teams ( National Integrated Care Programme Older Persons, 2021). Listed below ( Table 1) are some of the key mechanisms for team self-evaluation of performance identified in the framework and operational guidance:

**Table 1.** Key mechanisms for team self-evaluation of performance identified in the ECLECTIC framework ( O’Donnell *et al.*, 2021; O’Donnell *et al.*, 2022) and the HSE’s operational guidance for older person community-based multi-disciplinary teams ( National Integrated Care Programme Older Persons, 2021).

Understanding roles	• Professional role descriptors are available for each team member • Knowledge of each team members’ disciplinary expertise and competences • Knowledge of team vision and values • Frequency and attendance at team meetings • Trust *
Making referrals	• A standard operating procedure for making referrals is agreed upon and developed by the team • A service directory (listing the services available in the area) is accessible to all team members and is regularly updated • When receiving a referral each team member receives accurate information relevant to their function • Number of duplicate, declined and/or missed opportunities for referrals
Sharing information	• The older person’s preferences and consent regarding sharing information (who to share with and in what manner) is documented and reviewed regularly • Every older person (or family carer as appropriate) has a copy of their assessment and care plan, their discharge letter as well as any written documentation between HSCPs • A standardised dossier has been co-designed by the team for recording information that can be shared (with consent) and is accessible as a shared document
Communicating effectively	• Consistent utilisation of the older person’s preferred methods of communication (for example, verbal, written or communication aid) • A key worker is identified for any given case. The older person (or family carer where relevant) is aware of who their key worker is • A standard is agreed for timing of a response to an interprofessional referral/communication • Conflict *
Supporting the decision-making of older people	• The older person’s will, preferences and values for care planning and decision-making have been ascertained and are documented in an accessible file. • The older person’s will, preferences and values are identified and discussed in all care planning conversations • Where clinical and care planning decisions are made, they are explicitly shared with the older person’s will, preferences and values
Collective clinical decision-making	• Issue-specific decision-making is led by the team member with the most professional competence for that issue • Psychological safety within and across the team *

*Where possible, previously validated, and reliable instruments will be used to measure critical concepts associated with interprofessional collaboration in teams. Psychological safety will be measured using a validated tool developed by O’Donovan *et al.* (2020). Trust in teams will be measured using the tool developed and validated by Costa & Anderson (2011). A key objective of the exploratory scoping of the evidence in work package one will be to identify further robust and validated tools for measurement of outcomes that may be associated with and/or indicators of the effective interprofessional team working in healthcare including team conflict, work satisfaction, a sense of meaning and purpose for one’s role.

Other measures that may emerge from the evidence scoping and realist synthesis include attitude towards interdisciplinary working, knowledge of team roles, knowledge of each competency, trust, conflict, collective leadership/decision-making and sense of work engagement such as feelings of vigour, dedication and absorption. The measurements will be conducted online through individual scaled questionnaires. Previously validated and robust instruments will be used to measure outcomes, where available.

***Data collection: Two case study sites***

In collaboration with the project advisory committee, two of the nominated CST-OPs will be identified for more extensive data collection and realist evaluation. The following additional sources of data will be collected from these two teams. This will ensure the generation of rich and detailed explanatory programme theory for two case study sites. The data collected from the other sites will be used to support and refine the two case study programme theories.

**Observations of team meetings**

Structured observations of a team meeting will occur at three timepoints (baseline (T1), 6 months (T2), 12 months (T3) as above). The focus of these team observations will be to identify the contextual conditions and related mechanisms which lead to outcomes associated with interprofessional collaboration. A bespoke observation template will be developed to support data collection to promote consistency in data capture across observations. This will be modelled on a tool developed by a member of the research team with colleagues (McAuliffe) for observation and measurement of psychological safety in healthcare teams ( O’Donovan *et al.*, 2020). The observation tool will record the use of resource mechanisms such as the utilisation of standard operating procedures, team strategy documents as well as reasoning mechanisms such as actions, behaviours, body language and tone. The focus of analysis will be the relationship between generative contexts for these mechanisms and any associated outcomes.

**Documentary analysis**

Documentary analysis of team strategy and policy statements and files will be undertaken to support the generation of contextual data as well as the identification of resource mechanisms for developing competence, in particular examination of the team vision and mission statements (if available) as well as standard operating procedures for interdisciplinary team communication and referral processes. The teams will be asked to record adverse events and indicators of care quality including episodes of missed care, duplication of care, declined care (including assessment and referral). These will be recorded by the team in anonymised format and will be collected for the purposes of tracking frequency of adverse events over the observation time frame (12 months).

***Data analysis***

Qualitative data analysis of the interview transcripts, the documents and the team observational notes will be conducted using NVIVO Pro 12 Software (See Weft QDA for open-source alternative software for qualitative data analysis). Qualtrics XM will be used for the collection of quantitative data and data will be analysed using IBM SPSS Statistics 27. The analysis will be undertaken on an ongoing basis whereby each wave of data collection will iteratively inform the next wave of data collection in each case study site. Data analysis will be guided by Gilmore *et al.*’s (2019) methodological outline for realist evaluation and will adopt a retroductive approach which uses ‘both inductive and deductive reasoning and includes researcher insights to understand generative causation, by exploring the underlying social and psychological drivers identified as influencing programme outcomes’ ( Gilmore *et al.*, 2019).

The focus of data analysis in this second work package will be to observe outcome patterns to inductively identify C-M-O configurations and to deductively test IPTs within each of the two case study sites identified for in-depth realist evaluation. The outcome from this work package will be a refined programme theory for each of the two case study sites nominated for in-depth realist evaluation. The additional case study data from the other two sites will be used to support or refine the theories. At the end of the evaluation period (12 months) data will be reported to each of the case study sites. This will ensure that the knowledge generated from the evaluation can inform the study sites and be used to improve their interprofessional collaboration.

### Work package three: Programme theory refinement and consolidation and knowledge sharing

The third and final work package will involve the synthesis and sharing of the refined programme theories which emerged from the second work package across case study sites to develop a middle range theory. This is a higher order theory involving a synthesis of more granular programme theories to provide hypotheses about how different types of interventions might work in different types of contexts in realising overall programme outcomes ( Pawson & Tilley, 1997). The methodological guidance developed by Gilmore *et al.* (2019) will inform this process.

***Middle range theory development***

Findings from each case study will be collated alongside all their support evidence. The first step of analysis will involve combining the programme theories and C-M-O configurations from both cases. Commonalities within the combined PTs and C-M-O configurations will be identified and grouped into a framework. The next step of analysis will be to identify predictable patterns occurring across the data. The additional qualitative data extracted from all four case study sites will be deployed at this stage. Data from all four sites will be reviewed to identify explanatory information to facilitate the synthesis of patterns of causation. This will support and refine the theories that had arisen from each of the two case studies and help manage any discrepancies between the case study findings.

In line with realist methodology, the focus of this analysis will be to develop an understanding of the generative causality between different contexts and the mechanisms which are linked to specific outcomes ( Pawson & Tilley, 1997). The resulting middle-range programme theory will lead to a detailed explanation of the dynamic relationship between varying C-M-O configurations. The realist synthesis of the literature undertaken in work package one will help to provide additional context and interpretation for the emerging middle range theory and may add additional explanations for causal mechanisms. Specifically, this middle-range theory will define what works in building proficiency for interprofessional collaboration (mechanisms) and under what circumstances (contexts).

Three final validation workshops will be held with the four key stakeholder groups (HSCPs, operational leads, policymakers and clinical design managers, and PPRs). The purpose of these workshops will be to refine and validate the theories and to generate discussion on how this theory can support the workforce capacity development with specific reference to teaching and learning approaches.

***Translation of the middle range theory into operational guidance and curriculum development***

The realist evaluation proposed for this application will identify guiding principles that can be applied in practice to support effective interprofessional collaboration in the delivery of quality integrated care for older people in the community setting. Through dissemination, it will offer practical guidance and insights for policymakers and health and social care providers as well as organisational leaders, and innovators to support successful implementation programmes across the health systems nationally and internationally.

The proposed project will impact the national healthcare workforce planning and development and through the dissemination of outputs will influence international conversations concerning workforce capacity in health and social care. The implementation evaluation of the ECLECTIC framework will directly influence the development of professional standards and guidelines for clinical design to support the implementation of the national older person’s service model. This will inform the recruitment strategy for the programme managers as well as continuing professional development for the integrated care teams established through the programme.

We will work with the NCPOP to scale up the findings across the clinical programme for older people as well as translate these findings to other programmes which have been restructured to include a focus on interdisciplinary teams.

The next element of work package three will involve the translation of the middle range programme theory into an interdisciplinary curriculum for the education and training of health and social care professionals. This will involve revision of the ECLECTIC framework ( Figure 1) to include enhanced operational guidance and an implementation strategy for scale-up and spread across services involved in the care of older people, as represented within the NCPOP model of care.

A cross-disciplinary and institutional project team will be convened with representation from core health professional programmes (HCSPs, medicine, pharmacy, and nursing) as well as regulatory bodies (NMBI, CORU, Medical Council). The team will be supported by collaborators from the Australian Institute for Health Innovation at Macquarie University who will enable knowledge translation for broader health system impact. The team will also invite public and patient representatives from our PPI advisory group to become members. The aim of this project team is to co-design a curriculum framework which will map the knowledge and skills required for interprofessional collaboration. This framework will enhance interdisciplinary teaching, learning and assessment of interprofessional collaborative practice across health and social care professional curricula. It will be a resource for educators across health professional programmes as well as continuing professional development. It will support incremental learning of the appropriate knowledge and skills as well as provide recommendations for specific learning activities to scaffold competency among students. The middle range theory will be embedded into the framework ensuring that the curriculum is context specific and aligned to the mechanisms associated with measurable outcomes for interprofessional collaborative practice. This framework will be used to foster interdisciplinarity in the health and social care professional curricula and will form the basis for structured elective module development at both undergraduate and taught graduate levels.

## Patient and public involvement

Members of the research team are academic champions of PPI as part of the UCD PPI Ignite program. The team have long established experience enabling patient, public and practitioner involvement in health system change such as co-designing frailty pathways in acute care settings ( O’Donnell *et al.*, 2019) and promoting assisted decision-making in acute settings for care planning ( O’Donnell *et al.*, 2018). The team believes that meaningful public and patient involvement not only ensures the research quality and relevance, but it also ensures that our work is informed by broader democratic values and principles of accountability and transparency which are at the core of enabling person-centred integrated care. All PPI contributors will be adequately supported to participate. This includes financial compensation for their time as well as for out-of-pocket costs. A project link and PPR coordinator will be established to support communication with the PPRs and to act as an informal liaison.

The proposed study builds on a previous HRB-funded applied partnership project in which we co-designed with older people the ECLECTIC framework ( O’Donnell *et al.*, 2021; O’Donnell *et al.*, 2022). Four of the public and patient representatives of older people who co-designed the framework have contributed to the development of the realist evaluation proposal. These four public and patient representatives of older people have agreed to remain involved with this project and will form a project PPI Advisory Committee. Two family carers of older people will be nominated by Family Carers Ireland to join the PPI advisory committee upon commencement of this study.

Nongovernmental collaborators from Age Friendly Ireland and Family Carers Ireland will be members of the project steering committee. They will advise the research team on methodology and governance for the project. They will provide strategic direction as to the recruitment of older people and family carers for the PPI advisory committee. They will also advise on recruitment and data collection processes for the semi-structured interviews with older people in work package two. At the end of work package three, at the final validation workshop with the PPI advisory committee, the group will discuss the professional development of health and social care professionals in relation to interprofessional working with specific reference to education and training. They will also be invited to participate in a project team whose role is to co-design an interdisciplinary teaching and learning curriculum to increase competencies for interprofessional collaboration in the integrated care of older people.

## Study status

The work package one has commenced. The research team have completed an exploratory scope of the evidence and is in the process of completing workshops with the four key stakeholder groups to develop initial programme theories that will be explored and developed through the literature synthesis. Search strategy development for the retrieval of research evidence for the realist review and synthesis is underway.

## Dissemination of results

The engagement of key leaders from the NCPOP, as well as public and patient representatives as co-applicants and collaborators for this proposal, is a deliberate strategy to aid knowledge translation and dissemination. These key champions will enable the immediate translation of evidence generated from this project into the operational policy and practice of all CSTs for older people.

The National Clinical Advisor and Group Lead (NCAGL) Older Persons Office, the HSE and the NCPOP will play a critical role in supporting the translation of the findings across the national integrated care programmes generally. The inclusion of the CST-OP clinical leads is critical for the translation of the findings into clinical design and practice as well as the operational guidelines and procedures within the National Clinical Programme for Older People. Associate Professor Harrison, a collaborator for this project, leads the healthcare engagement and workplace behaviour stream at the Australian Institute of Health Innovation, Macquarie University. The institute is focused on related areas of health services research, health informatics and health systems and safety. Harrison will work with co-applicant Ní Shé who has an honorary appointment at the institute in enabling knowledge translation of the work in Australia.

The research team will be in the UCD Centre for Interdisciplinary, Research, Education and Innovation (UCD IRIS) and will harness the knowledge and expertise of the centre in translating study findings into broader health systems research and education. This will ensure that the knowledge generated from this study will have immediate relevance for future workforce capacity development and ongoing professional education. This translation into ongoing professional development will also be facilitated through the creation of learning resources for integrated care teams that will be developed in collaboration with the HSE and NCPOP as a result of this study.

The findings from this study will also be disseminated to the international research community through publications in open-access peer-reviewed journals as well as presentations in national and international conferences. UCD Library, as a member of the IReL consortium of Irish academic libraries, has entered into a few Open Access Publishing agreements with key scholarly publishers. The budget request for this study will also provide for publication of two research papers in international open access peer-reviewed journals. Finally, this study protocol and a final study summary will be submitted for publication in HRB Open Research.

The results of the realist review will be reported according to the Realist and Meta-narrative Evidence Synthesis: Evolving Standards (RAMESES) quality and publication standards ( Wong *et al.*, 2016). Project quantitative data outputs and survey will be archived in the Irish Social Science Data Archive (maintained by UCD Library) which is Ireland’s leading centre for quantitative data acquisition, preservation, and dissemination. We will deposit the qualitative data and interview guides from this project with the Digital Repository of Ireland. A DOI will make the data findable and rich metadata will be provided to describe the data –the data will be clearly licenced to ensure researchers know what kind of reuse is permitted.

## Discussion

The health system reform in Irish national policy identified the need for a team-based approach to implementing integrated health and social care services successfully. The team-based approach also underpins the successful delivery of the NCPOP model of care. The core competencies necessary for interprofessional collaboration in CSTs for older people are outlined in the ECLECTIC framework ( O’Donnell *et al.*, 2021; O’Donnell *et al.*, 2022). However, a formal assessment and understanding of how team working can be enhanced and supported in older people’s care are underdeveloped compared with other specialist health areas ( Ellis & Sevdalis, 2019). The project will adopt a realist-informed process evaluation of how CSTs integrating care for older people adopt and adapt the ECLECTIC framework to develop core competencies for interprofessional collaboration. The evaluation will involve an implementation evaluation of the competencies as they are fostered in the newly developed CST-OPs.

Realist evaluations are increasingly applied to evaluate complex healthcare interventions due to their ability to provide a more explicit and in-depth understanding of what works, for whom and in what circumstances in relation to a particular intervention ( Nurjono *et al.*, 2018). Pawson & Tilley (1997) argue that an intervention increases its chances of successful outcomes if the appropriate mechanisms are applied to the right context with appropriate social and cultural conditions. By adopting a realist approach, findings from this study are expected to generate contextually relevant evidence for improving interprofessional collaboration among interdisciplinary integrated care teams for older persons and consequently improve health outcomes for older people.

Realist methodology encourages the involvement of relevant stakeholders in the design of the evaluation and interpretation of findings. This approach assists in defining evaluation questions, objectives and outcomes as well as understanding the implementation context of the programmes and how interventions have been delivered. This ensures that the evaluation will be rigorous, produces relevant insights for programme improvement, and provides evidence to support policy decisions ( Nurjono *et al.*, 2018). Early engagement of stakeholders in the evaluation process will increase the relevance of the study findings as well as the likelihood that study recommendations will be adopted.

The proposed realist evaluation will generate evidence regarding the outcomes associated with interprofessional collaboration in the care of older people. Furthermore, it will provide a nuanced realist understanding of the influence of contextual conditions in enabling or inhibiting mechanisms that foster, enhance, and sustain interprofessional collaboration. This explanatory theory will expand the ECLECTIC framework with an understanding of what works in supporting interdisciplinary teams to develop competency in interprofessional collaboration, for whom it works, in what contexts, and how.

This understanding will underpin curriculum development for team-based education and training of HSCPs, a key priority area in the national health strategy. It will also inform future international research by providing a foundational theory to support the exploration of how interdisciplinary teams across multiple settings, specialities, and contexts can build competencies for interprofessional collaboration.

## Data Availability

No data are associated with this article.
