# Supplementary material for: A realist process evaluation of an intervention to promote competencies in interprofessional collaboration among interdisciplinary integrated care teams for older people: Study protocol
Source: HRB Open Res. 2023 Sep 13;6:49. [Version 1] doi: 10.12688/hrbopenres.13729.1 (PMC10579852; doi:10.12688/hrbopenres.13729.1)
Supplement: Peer review history

# Reviewer report: Reviewer response for version 1

**DOI:** 10.21956/hrbopenres.15017.r36449

**Published:** 2023-11-01

**Authors:** Kathryn Lizbeth Siongco

**Affiliations:** 1 College of Nursing, University of the Philippines Manila, Manila, Metro Manila, Philippines

The study clearly illustrated the method of generating new knowledge on how interprofessional collaboration can be successfully integrated and sustained in contexts involving the care of older persons. 

 However, there are some points to be improved further.

 First is the use 'interprofessional' instead of 'interdisciplinary'. The authors must be aware that a stark difference exist between the two terms and consistency must be reflected in the paper.

 Second, the ECLECTIC framework was described to revolve around person-centered values on top of the 6 domains of IPC competencies. As I understood correctly, the IPC competencies (figure 1) revolved around person-centered values but this was not reflected in the figure.

 Third, when talking about IPC team members, it is relevant to provide a description of the health professionals involved and their roles in the team considering the healthcare landscape in Ireland.

Is the study design appropriate for the research question?

Yes

Is the rationale for, and objectives of, the study clearly described?

Yes

Are sufficient details of the methods provided to allow replication by others?

Yes

Are the datasets clearly presented in a useable and accessible format?

Not applicable

Reviewer Expertise:

Ageing, inteprofessional education and collaborative practice, quantitative research methods

I confirm that I have read this submission and believe that I have an appropriate level of expertise to confirm that it is of an acceptable scientific standard, however I have significant reservations, as outlined above.

# Reviewer report: Reviewer response for version 1

**DOI:** 10.21956/hrbopenres.15017.r36447

**Published:** 2023-10-16

**Authors:** Sherry Dahlke

**Affiliations:** 1 Faculty of Nursing, University of Alberta, Edmonton, Alberta, Canada

This is an excellently written paper outlining an extensive process to improve care of older people. Starting out by conducting a realist review will situate the subsequent study of 4 cases quite well. I appreciate the conducting of interviews and observations of team meetings initially, in six months and in a year as an important way to see differences in how teams develop and demonstrate competencies. Developing the mid-range theory to support how the competencies are applied to older adult care over different contexts will support the end goal of developing curriculum for interprofessional education. I look forward to further publications as this process unfolds

Is the study design appropriate for the research question?

Yes

Is the rationale for, and objectives of, the study clearly described?

Yes

Are sufficient details of the methods provided to allow replication by others?

Yes

Are the datasets clearly presented in a useable and accessible format?

Yes

Reviewer Expertise:

Nursing care of older adults, nursing education and interprofession practice

I confirm that I have read this submission and believe that I have an appropriate level of expertise to confirm that it is of an acceptable scientific standard.

# Reviewer report: Reviewer response for version 1

**DOI:** 10.21956/hrbopenres.15017.r36127

**Published:** 2023-09-29

**Authors:** Rosa McNamara

**Affiliations:** 1 Emergency Department, St. Vincent University Hospital, Dublin, Ireland

This paper describes the methodology for a realist review to evaluate the implementation of the ECLECTIC framework for the development of competencies in interdisciplinary community-based teams for older people.

 The goals are clearly described with an appropriate methodology clearly described and suitable to answer the research question.

 There is a structured plan of work packages that is realistic and deliverable.

 The methodology clearly describes how different sources of information will be identified and used to inform the research.  In particular, the explanation of the chosen methodology and rational for this choice is clearly presented.

 The plan for ethics review is appropriate.

 The plan for dissemination of results is also appropriate and hopes to influence policy meaningfully as well as to share learning from the research.

Is the study design appropriate for the research question?

Yes

Is the rationale for, and objectives of, the study clearly described?

Yes

Are sufficient details of the methods provided to allow replication by others?

Yes

Are the datasets clearly presented in a useable and accessible format?

Not applicable

Reviewer Expertise:

Geriatric Emergency Medicine, Medical Education

I confirm that I have read this submission and believe that I have an appropriate level of expertise to confirm that it is of an acceptable scientific standard.
